# Supplementary material for: The preventive and therapeutic effects of probiotics on mastitis: A systematic review and meta-analysis
Source: PLoS One. 2022 Sep 9;17(9):e0274467. doi: 10.1371/journal.pone.0274467 (PMC9462749; doi:10.1371/journal.pone.0274467)
Supplement: S1 Table — (DOCX) [file pone.0274467.s001.docx]

| **Database**  **S1 Table.** Search strategy. | **Search query** | **Results** |
| --- | --- | --- |
| **PubMed** | #1∙ "mastitis"[MeSH Terms] OR "mastitis"[Title/Abstract] OR "mammary inflammation"[Title/Abstract] OR "mastadenitis"[Title/Abstract] OR "breast infection"[Title/Abstract] OR "breast inflammation"[Title/Abstract] OR "mammary infection"[Title/Abstract]  #2∙ "probiotics"[MeSH Terms] OR "probiotic*"[Title/Abstract] OR "prebiotic*"[Title/Abstract] OR "synbiotic*"[Title/Abstract] OR "lactobacillus"[Title/Abstract]  #3∙ #1 AND #2 | **122** |
| **Cochrane Library** | #1∙ MeSH descriptor: [Mastitis] explode all trees  #2∙ (mastitis):ti,ab,kw OR (''breast infection''):ti,ab,kw OR (''breast inflammation''):ti,ab,kw OR (''mammary inflammation''):ti,ab,kw OR (''mammary infection''):ti,ab,kw  #3∙ #1 or #2  #4∙ MeSH descriptor: [Probiotics] explode all trees  #5∙ (probiotic*):ti,ab,kw OR (prebiotic*):ti,ab,kw OR (synbiotic*):ti,ab,kw OR (lactobacillus):ti,ab,kw  #6∙ #4 or #5  #7∙ #3 and #6 | **126** |
| **Embase** | #1∙ 'mastitis'/de OR mastitis:ab,ti OR 'breast infection':ab,ti OR 'breast inflammation':ab,ti OR 'mammary infecton':ab,ti OR 'mammary inflammation':ab,ti  #2∙ 'probiotic agent'/de OR probiotic*:ab,ti OR prebiotic*:ab,ti OR synbiotic*:ab,ti OR lactobacillus:ab,ti  #3∙ #1 AND #2 | **208** |
| **Web of Science** | #1∙TS=(mastitis or “breast infection” or “breast inflammation” or “mammary infection” or “mammary inflammation” )  #2∙ TS=(probiotic* or prebiotic* or synbiotic* or lactobacillus)  #3∙ #2 AND #1 | **304** |
| **CNKI** | (SU='乳腺炎' OR SU='乳痈' OR SU='乳房炎症' OR SU='乳房感染') AND (SU='益生菌' OR SU='益生元' OR SU='有益菌' OR SU='益生素' OR SU='活菌制剂' OR SU='微生态制剂' OR SU='乳酸菌' OR SU='乳杆菌') | **21** |
| **Wanfang** | （（主题：“乳腺炎”）OR（主题：“乳痈”）OR（主题：“乳房炎症）OR（乳房感染”）） AND （（主题：“益生菌”）OR（主题：“益生元”）OR（主题：“有益菌”）OR（主题：“益生素”）OR（主题：“活菌制剂”）OR（主题：“微生态制剂”）OR（主题：“乳酸菌”）OR（主题：“乳杆菌”）） | **26** |
| **Total** |  | **807** |
