# Supplementary material for: The preventive and therapeutic effects of probiotics on mastitis: A systematic review and meta-analysis
Source: PLoS One. 2022 Sep 9;17(9):e0274467. doi: 10.1371/journal.pone.0274467 (PMC9462749; doi:10.1371/journal.pone.0274467)
Supplement: S2 Table — (DOCX) [file pone.0274467.s002.docx]

**S2 Table:** Characters of related ongoing studies

| **Identifier** | **Study title** | **Registration year** | **Target size** | **Inclusion criteria** | **interventions** | **locations** | **Outcomes measures** | **status** |
| --- | --- | --- | --- | --- | --- | --- | --- | --- |
| **NCT04032899** | Multicenter, Randomized, Double-blind, Controlled Parallel Nutritional Intervention Study to Evaluate the Effect of Consumption During Pregnancy and the Lactation Period of Lactobacillus Fermentum CECT5716 on the Incidence of Mastitis | 2019 | 480 | Normal development of pregnancy;  Single fetus pregnancy;  Be in week 28-32 of pregnancy;  Intention to breastfeed the child for 16 weeks | Lactobacillus fermentum CECT5716/Maltodextrin | Spain | Incidence of mastitis;  Microbiota of breast milk;  Recurrence of mastitis;  Breast pain questionnaire;  Time of Breastfeeding;  Percentage of infants breastfeed;  Measurement of bio-markers of inflammation, minerals and immunoglobulins in breast milk;  Baby feces microbiota and anthropometric measures;  incidence of cesareans and incidence of antibiotic use during delivery;  Data about the intestinal health and sleep parameters of the baby | Recruiting |
| **IRCT20100414003706N38** | Effect of probiotic supplementation for mother and for infant on bilirubin level and weight gain in very low birth weight infants: a three parallel arm randomized controlled trial | 2021 | 75 | Delivery in the past 48 h  Desire and ability to breastfeed her baby  Neonatal birth weight of 1000 to 1500 g  Hospitalization of the infant for at least 7 days after the start of the intervention  Possibility of the mother to attend hospital where the baby is admitted, at least once a week | Mother: probiotic supplement containing 1.5× 10 ^9^ CFU of Lactobacillus Paracasei/placebo | Iran (Islamic Republic of) | Total serum bilirubin level;  Weight gain during neonatal period;  Composite variable of occurrence of serious neonatal problems;  Duration of infant hospitalization;  Infant age at full enteral feeding (day);  Length of total parenteral nutrition;  Occurrence of mastitis in the mother;  Total duration of phototherapy (hours) | Recruiting |
| **NTR4388** | A randomized, double-blind, placebo-controlled intervention study to assess the preventive effect of new probiotic strain on lactational mastitis | 2014 | 300 | Healthy pregnant, adults (> 18 years of age);  Before/during the 35th week of pregnancy;  Intending to breastfeed her infant;  Written informed consent. | probiotic supplement/placebo | The Netherlands | Incidence (hazard) rate of mastitis;  Count of recurrent episodes of mastitis;  Incidence (hazard) rate of breastfeeding withdrawal (complete/partial discontinuation) | The results have not yet been published |
| **ACTRN12615000923561** | Evaluation of the probiotic Lactobacillus Fermentum CECT5716 for the prevention of mastitis in breastfeeding women: a randomized controlled trial | 2015 | 600 | Pregnant women at least 18 years of age at 37 weeks’ gestation or later with a singleton pregnancy will be invited to participate in the trial. They will currently not be taking commercial probiotics containing lactobacillus fermentum; and will own a smartphone. Their intention at the time of consent will be to breastfeed their baby. | probiotic sachets containing Lactobacillus Fermentum (1x10^10^ CFU/mL)/placebo | Australia | incidence of mastitis;  Length of time breastfeeding;  Breastfeeding complications;  Overall maternal health and well-being assessed by patient surveys;  Acceptability and compliance of trial using a mobile phone application system;  Preference for method of postnatal questionnaires;  Infant health and well-being measured | The results have not yet been published |
