# Supplementary figures and images for: The preventive and therapeutic effects of probiotics on mastitis: A systematic review and meta-analysis
Source: PLoS One. 2022 Sep 9;17(9):e0274467. doi: 10.1371/journal.pone.0274467 (PMC9462749; doi:10.1371/journal.pone.0274467)

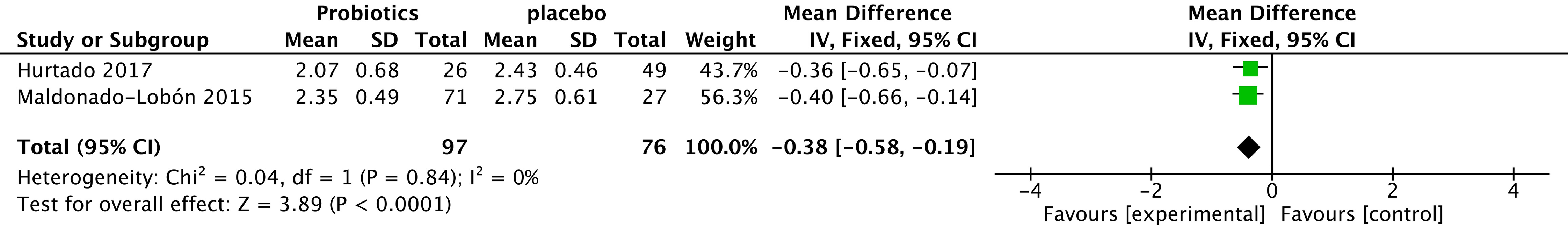

Supplement: S1 Fig — (TIF) [file pone.0274467.s004.tif]

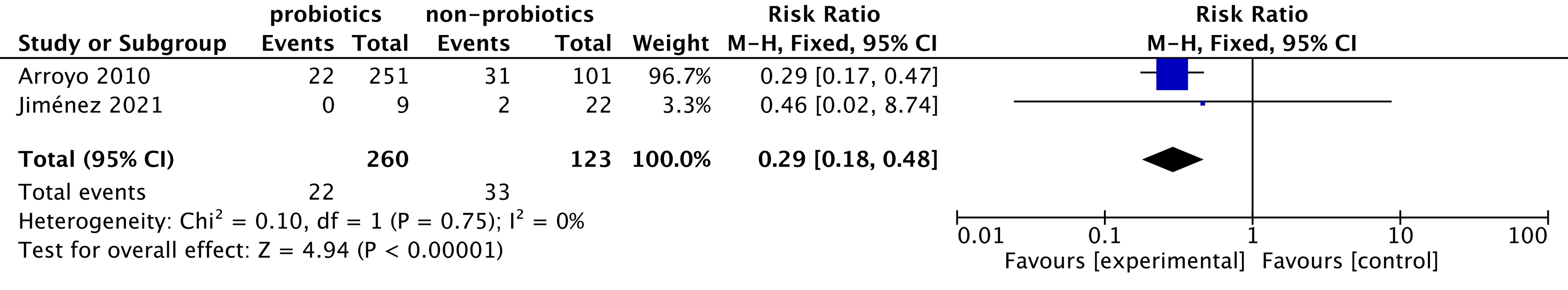

Supplement: S2 Fig — (TIF) [file pone.0274467.s005.tif]
