# Supplementary material for: The preventive and therapeutic effects of probiotics on mastitis: A systematic review and meta-analysis
Source: PLoS One. 2022 Sep 9;17(9):e0274467. doi: 10.1371/journal.pone.0274467 (PMC9462749; doi:10.1371/journal.pone.0274467)
Supplement: Attachment — Submitted filename: PONE-D-21-38695_reviewers comment.pdf [file pone.0274467.s006.pdf]

The preventive and therapeutic effects of probiotics on mastitis: A Systematic Review and Meta-Analysis

Qinghong Yu · Chuchu Xu · Mengqian Wang · Jiayan Zhu · Linghong Yu · Zimei, Yang, Shan Liu and Xiufei Gao*

Yu et al, in this study, meta-analyzed data to identify effects of probiotics on the prevention and treatment of human mastitis. The data of participants of randomized control treatment studies included healthy pregnant and lactating woman taking probiotics systematically by oral route during pregnancy and lactation. Meta-analysis of six data showed that oral doses of prebiotics significantly reduced the incidence of mastitis and tendency of reduction of breast pain rate. Of these, two studies showed highly significant reduction in milk somatic cell count in two studies of healthy woman and five studies of mastitis patients. Increased number of milk somatic cell count is a definite maker of mastitis. It was concluded that, to a certain extent, probiotic intake appears to reduce incidence of mastitis and mastitis-related symptoms in pregnant and lactating woman.

Though, this study explored 807 records, only six studies qualified for inclusion in this review. Due to lack of sufficient data, role of probiotics in reducing mastitis and mastitis associated symptoms are speculative. Though, authors have acknowledged the limitations of study and suggest high-quality multicenter trails to support their results.

Major issues

1. Line 45: A total of six studies included in the meta-analysis. However, later it is mentioned that two studies of healthy people and five studies of mastitis patients, a total of seven studies are discussed. Please explain this discrepancy or make it the statement clearer.

2. Line 46: "……during pregnancy can reduce the incidence of mastitis (RR: 0.49, 95%CI: 0.35 to 0.69; $p<0.0001$) and breast pain rate (RR: 0.81, 95 %CI: 0.64 to 1.01; $p=0.07$)". This statement explains reduction ($P < 0.05$) in breast pain rate, which is in fact a tendency of reduction in the pain rate ($P = 0.07$). Please explain or make the necessary changes.

Minor issues

- Please be consistent with using word "Figure" or "Fig." See the guidelines of PlosOne.
