# Supplementary material for: The preventive and therapeutic effects of probiotics on mastitis: A systematic review and meta-analysis
Source: PLoS One. 2022 Sep 9;17(9):e0274467. doi: 10.1371/journal.pone.0274467 (PMC9462749; doi:10.1371/journal.pone.0274467)
Supplement: Attachment — Submitted filename: Response to Reviewers.docx [file pone.0274467.s007.docx]

Dear editors and reviewers:

Thank you for your letter and comments on our manuscript titled “The preventive and therapeutic effects of probiotics on mastitis: A Systematic Review and Meta-Analysis” (PONE-D-21-38695). These comments helped us improve our manuscript, and provided important guidance for future research.

We have addressed the editor’s and the reviewers’ comments to the best of our abilities, and revised text to meet the PLOS ONE style requirements. We hope this meets your requirements for a publication.

Since our original manuscript has been professionally polished, there may be some differences in wording from my previous manuscripts, but we did not change the original intention. Relevant revisions made in response to reviewer comments, we marked the revised portions in red and highlighted them yellow in manuscript. The main comments and our specific response are detailed below:

**Reviewer 1:**

**1. Line 45: A total of six studies included in the meta-analysis. However, later it is mentioned that two studies of healthy people and five studies of mastitis patients, a total of seven studies are discussed. Please explain this discrepancy or make it the statement clearer.**

Response：I am sorry that this part was not clear in the original manuscript, I should explain that during the review process, we included a total of 6 studies, 2 of which included double data of healthy people and patients with mastitis, and the data discussed at the end were all from these 6 studies. My language did not express clearly, the expression has been removed in the revised manuscript.

**2. Line 46: “……during pregnancy can reduce the incidence of mastitis (RR: 0.49,**

**95%CI: 0.35 to 0.69; p<0.0001) and breast pain rate (RR: 0.81, 95 %CI: 0.64 to 1.01; p=0.07)”. This statement explains reduction (P < 0.05) in breast pain rate, which is in fact a tendency of reduction in the pain rate (P = 0.07). Please explain or make the necessary changes.**

Response: Thanks for your suggestion, I have revised the abstract accordingly.

**3. Please be consistent with using word “Figure” or “Fig.” See the guidelines of PlosOne.**

Response: Thanks for your suggestion, I have made the corresponding changes according to the guidlines of PLOS ONE.

**Reviewer 2:**

**1.The manuscript has included just 6 studies which are not enough to draw a conclusion under the review.**

Response: Thank you for your suggestion. Due to the widespread abuse of antibiotics and the prudent choice of medication during pregnancy/lactation, our team believes that it is meaningful to evaluate the intervention effect of probiotics at this time. There are really not many relevant studies at present, and we have acknowledged this in the limitation of manuscript. However, since the relevant experiments in the registration have not been published, our research team is also conducting relevant experiments recently. We hope to supplement this evidence when follow-up related research results are available.

**2. The presentation of manuscript is not according to journals instructions/format.** **The abstract is not in proper format.**

Response: Thanks for your suggestion. I have already revised the format of our manuscript.

**3. The Materials and methods and conclusion parts need to be improved.**

Response: Thanks for your suggestion. Since we did not know the specific modification goals, we consulted Liu S, a statistician, again. And made corresponding changes in the materials and methods and conclusion parts, we hope that this revision will be better than before.

**Editors:**

Thanks for your suggestion, we have uploaded and all the figure files to the Preflight Analysis and Conversion Engine (PACE) digital diagnostic tool and passed the certification. So, we re-uploaded our figure file with no changes to the data.

We would be glad to respond to any further questions and comments that you may have.

Sincerely yours,

Xiufei Gao
